# *Phylo-mLogo*: an interactive and hierarchical multiple-logo visualization tool for alignment of many sequences

**DOI:** 10.1186/1471-2105-8-63

**Published:** 2007-02-24

**Authors:** Arthur Chun-Chieh Shih, DT Lee, Chin-Lin Peng, Yu-Wei Wu

**Affiliations:** 1 Institute of Information Science, Academia Sinica, Taipei, 115, Taiwan; 2 Genomics Research Center, Academia Sinica, Taipei, 115, Taiwan

## Abstract

**Background:**

When aligning several hundreds or thousands of sequences, such as epidemic virus sequences or homologous/orthologous sequences of some big gene families, to reconstruct the epidemiological history or their phylogenies, how to analyze and visualize the alignment results of many sequences has become a new challenge for computational biologists. Although there are several tools available for visualization of very long sequence alignments, few of them are applicable to the alignments of many sequences.

**Results:**

A multiple-logo alignment visualization tool, called *Phylo-mLogo*, is presented in this paper. *Phylo-mLogo *calculates the variabilities and homogeneities of alignment sequences by base frequencies or entropies. Different from the traditional representations of sequence logos, *Phylo-mLogo *not only displays the global logo patterns of the whole alignment of multiple sequences, but also demonstrates their local homologous logos for each clade hierarchically. In addition, *Phylo-mLogo *also allows the user to focus only on the analysis of some important, structurally or functionally constrained sites in the alignment selected by the user or by built-in automatic calculation.

**Conclusion:**

With *Phylo-mLogo*, the user can symbolically and hierarchically visualize hundreds of aligned sequences simultaneously and easily check the changes of their amino acid sites when analyzing many homologous/orthologous or influenza virus sequences. More information of *Phylo-mLogo *can be found at URL .

## Background

Sequence alignment and inference of the phylogenies is a standard procedure for analyzing biological sequences. Based on the reconstructed phylogenetic relationship, the evolutionary histories of sampled species, individuals, or strains can be inferred. Traditionally researchers are used to assigning numbers to all clades in the phylogenetic analysis of individual gene segments and using them to represent and compare genotypes across homologous genes or multiple species [1]. However, when the number of analyzed sequences is in the hundreds or thousands, this approach cannot distinguish the differences among sequences from different homologous genes [1,2] or from different strains, such as viral sequences from different strains [3]. Moreover, the evolutionary changes of some specific sites, such as positively selected or antigenic sites, cannot be directly observed by global phylogenetic analysis, short of checking the detailed alignment results. Therefore, how to design efficient tools for biologists to analyze and visualize alignments of many sequences has become a challenge for computational biologists.

In recent years there are several visualization tools of sequence alignments available in the public domain. Based on the visualization outputs, these tools can be divided into two categories: curve-based and sequence-logo-based. In the former category, tools, such as VISTA family [4], PipMaker [5,6], zPicture [7], and SinicView [8], were developed to visualize individual alignment results or to compare and evaluate assorted alignment results obtained by different tools. These tools are useful for visualizing very long sequence alignment results of a few sequences. However, for cases of many but short sequence alignments, they are impractical because some significant variations between sequences may be submerged by global scoring profiles which are calculated by either identical rates or sum-of-pair scores.

In the latter category, tools such as WebLogo [9] and LogoBar [10], were developed, using sequence logos to graphically represent informative patterns of each individual site in a multiple sequence alignment. The sequence logos can assist the user to discover and identify conserved patterns from multiple sequence alignments [11]. The original work was first proposed by Schneider and Stephens (1990). Crooks *et al*. (2004) developed WebLogo [9] and performed an extension that incorporates additional features and options. To distinguish the gaps and poorly conserved positions, LogoBar [10] was proposed to display protein sequence logos including both amino acids and gaps. These sequence-logo-based tools are very useful to globally visualize consensus patterns in a multiple sequence alignment. However, when the number of aligned sequences is in several hundreds or thousands, some significant local tendencies of mutations cannot be observed directly from these global sequence-logo-based profiles.

All of these tools can only generate a global sequence logo for one multiple sequence alignment and have no interactive user interface to allow the user to compare sequence logos in different clades simultaneously, to manipulate the comparison by various observation ranges and individual sites, or to generate a set of images or PDFs of multiple logos comparison. In the analysis of influenza virus evolution, for example, tracking the transitional changes of the amino acids at the epitope or receptor binding sites is very important, because their changes could cause antigenic drift [12], affect viral transcription [13], and determine receptor specificity [14]. Furthermore, identifying genetic signatures of natural selection in coding or other functional regions can help researchers examine functional and evolutionary characteristics of alignment regions [15,16]. To improve the capabilities of the above-mentioned tools and help researchers better understand influenza virus evolution or more easily identify genetic signatures we develop a multiple-logo alignment visualization tool, called *Phylo-mLogo*, which allows the user to simultaneously visualize the global profile of the whole multiple sequence alignment and homologous logos of each clade in a hierarchical manner. *Phylo-mLogo *calculates the variabilities and homogeneities of aligned sequences by base frequencies or entropies. Different from the traditional representations of sequence logos, *Phylo-mLogo *not only displays the global logo patterns of the whole alignment but also demonstrates their local logos for each clade. In addition, *Phylo-mLogo *also allows the user to focus only on the analysis of some important, structurally or functionally constrained sites in the alignment selected by the user or by built-in automatic calculation.

## Implementation

There are a viewing section and a panel control section in *Phylo-mLogo*. The viewing section includes Root Logo View, Clade Logos View, and Detailed Text Alignment View, while the control section provides the user with a suite of versatile control functions for visualizing the alignment results from different perspectives. In what follows, we will introduce the characteristics and functionality of *Phylo-mLogo *in more detail.

### Scoring methods in *Phylo-mLogo*

The scoring methods adopted in *Phylo-mLogo *to calculate the variabilities and homogeneities of aligned sequences are based on entropy or base frequencies. Sequence logos graphically represent informative patterns in a multiple sequence alignment [9,11]. Schneider and Stephens (1990) defined the sequence conservation *R*_*seq *_at a fixed position in the alignment as the difference between the maximum possible entropy *S*_*max *_and the entropy S_obs _of the observed symbol distribution [11]:

Rseq=Smax⁡−Sobs=log⁡2N−(−∑i=1Npilog⁡2pi),
 MathType@MTEF@5@5@+=feaafiart1ev1aaatCvAUfKttLearuWrP9MDH5MBPbIqV92AaeXatLxBI9gBaebbnrfifHhDYfgasaacH8akY=wiFfYdH8Gipec8Eeeu0xXdbba9frFj0=OqFfea0dXdd9vqai=hGuQ8kuc9pgc9s8qqaq=dirpe0xb9q8qiLsFr0=vr0=vr0dc8meaabaqaciaacaGaaeqabaqabeGadaaakeaacqWGsbGudaWgaaWcbaGaem4CamNaemyzauMaemyCaehabeaakiabg2da9iabdofatnaaBaaaleaacyGGTbqBcqGGHbqycqGG4baEaeqaaOGaeyOeI0Iaem4uam1aaSbaaSqaaiabd+gaVjabdkgaIjabdohaZbqabaGccqGH9aqpcyGGSbaBcqGGVbWBcqGGNbWzdaWgaaWcbaGaeGOmaidabeaakiabd6eaojabgkHiTiabcIcaOiabgkHiTmaaqadabaGaemiCaa3aaSbaaSqaaiabdMgaPbqabaaabaGaemyAaKMaeyypa0JaeGymaedabaGaemOta4eaniabggHiLdGccyGGSbaBcqGGVbWBcqGGNbWzdaWgaaWcbaGaeGOmaidabeaakiabdchaWnaaBaaaleaacqWGPbqAaeqaaOGaeiykaKIaeiilaWcaaa@5CC6@

where *p*_*i *_is the observed frequency of symbol *i *at a fixed position and *N *is the number of distinct symbols for the given sequences types [11]. The maximum sequence conservation per site is log_2_4 = 2 bits for nucleotide sequences and log_2_20 ≈ 4.32 bits for amino acid sequences [9]. A sequence logo shows each base by the total number of bits of information multiplied by the relative occurrence of the nucleotide or amino acid at the position. Sequence logos enable fast and intuitive visual assessment of pattern characteristics applied to many fields [17]. However, in some not highly conserved cases, the entropies of the sequences are usually lower or just close to half of maximum sequence conservation so that the generated sequence logos may be compressed together and not conspicuous. Thus, the magnitudes of the sequence logos in some literature reflect only the relative occurrence frequencies of the bases at the position, without being multiplied by their entropies [18,19]. For convenience of making a distinction, the latter type of sequence logos is called the *frequency sequence logos*, and the former type the *entropy sequence logos*. The user can select different type of sequence logos shown in the Root Logo View and Clade Logos View sections.

### Viewing section in Phylo-mLogo

**Figure 1:**
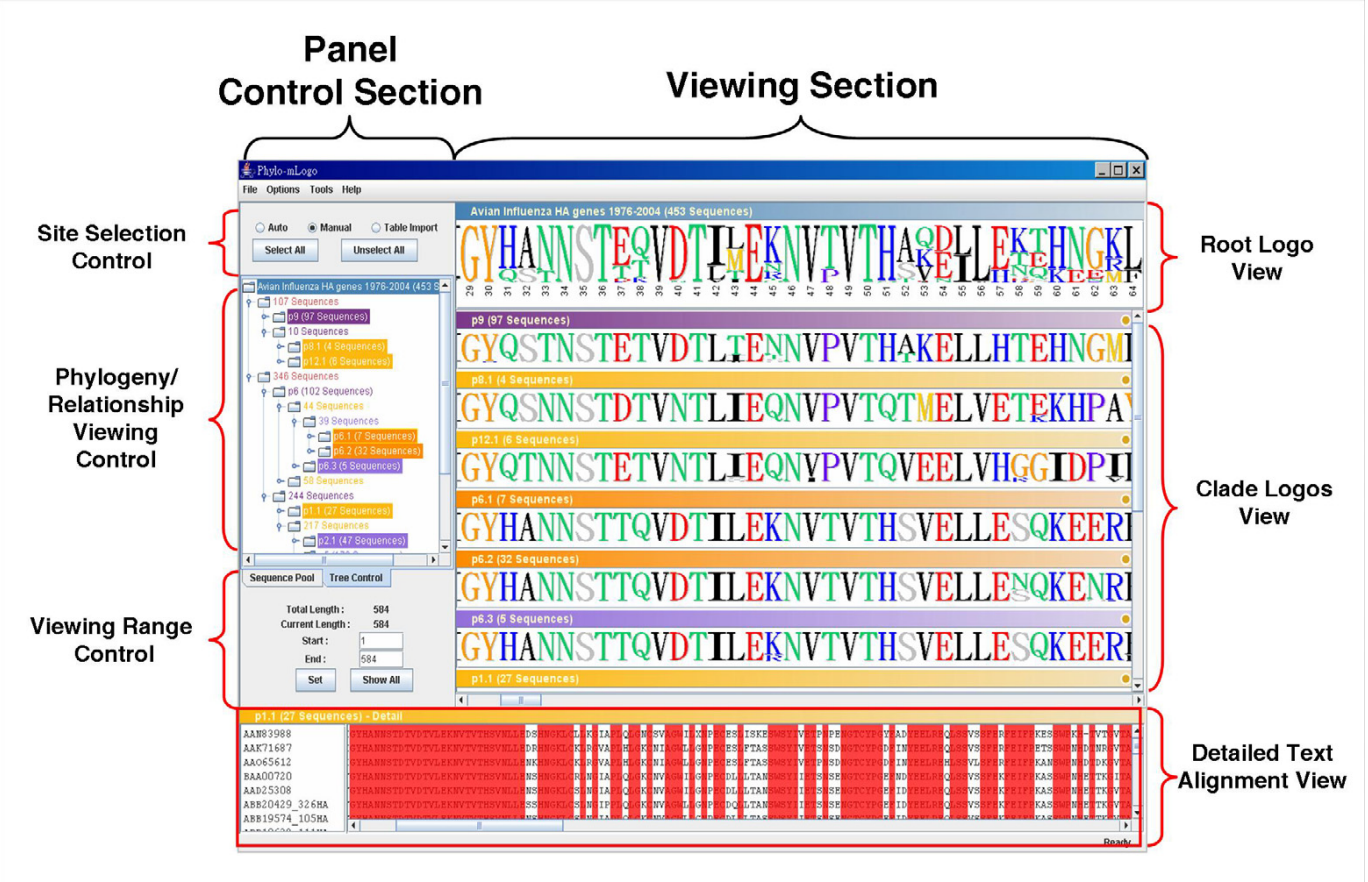
**The screenshot shows the user interface of *Phylo-mLogo***. The sequence logos of the influenza virus HA isolates from New York for the whole periods and different influenza seasons, Nov. 1998–Mar.1999, Oct.1999–Mar. 2000, Nov. 2001–Mar. 2002, Feb.–May. 2003, and Oct. 2003–Feb. 2004. In the Viewing Section, the sequence logo shown in Root Logo View represents the frequencies of the amino acids over the whole aligned sequences while those shown in Clade Logos Views demonstrate the logos for the sequences belonging to the same clade.

As shown in Fig. 1, the sequence logo in the Root Logo View represents the base information of the whole alignment and the heights of each logo are the occurrence frequencies. If the user selects the entropy option, all heights of the logos will be multiplied by their entropies. Below the logos in this View, the positions in the whole alignment are also marked so as to assist the user in comparison with others in the literature.

In the Clade Logos View, the panels show different sequence logos of the aligned sequences belonging to selected clades. By observing the graphical results, it is much more intuitive and straightforward to judge and identify the selective or evolutionary trends of alignment results between different phylogeny, regionalization, or seasonality. When the user clicks the colored bar above the logo graphics, the Detailed Text Alignment View will automatically show the detailed alignment result in a colored text format where identical characters are shown.

### Panel control section in *Phylo-mLogo*

In the control section, there are three panels for selecting the sequence logos from root to clades and the sites from totality to partiality: Phylogeny/Relationship Viewing Control, Site Selection Control, and Viewing Range Control. Phylogeny/Relationship Viewing Control shows the relationship structure of aligned input sequences. The structure could be the phylogenetic tree for cross-species or different lineage sequences, the regionalization for polymorphic sequences, or the seasonalities for epidemic influenza virus sequences. Because showing a scaled tree usually takes up a lot of space on a screen, Phylogeny/Relationship Viewing Control in *Phylo-mLogo *demonstrates these relationships by a hierarchical file system browser, an unscaled tree, in which each node (a clade) represents a group of well-aligned sequences and the related child nodes will be expanded once the user clicks the parent node. As a file or directory name shown in the file browser, there is a name following each node given by the user or by built-in automatic assignment. When the user clicks the name part of either a parent or child node, the sequence logo associated with this node will be calculated and the result shown in the Clade Logos View. When the user clicks again the name part, which is a toggle switch, its sequence logo will become invisible.

**Figure 2:**
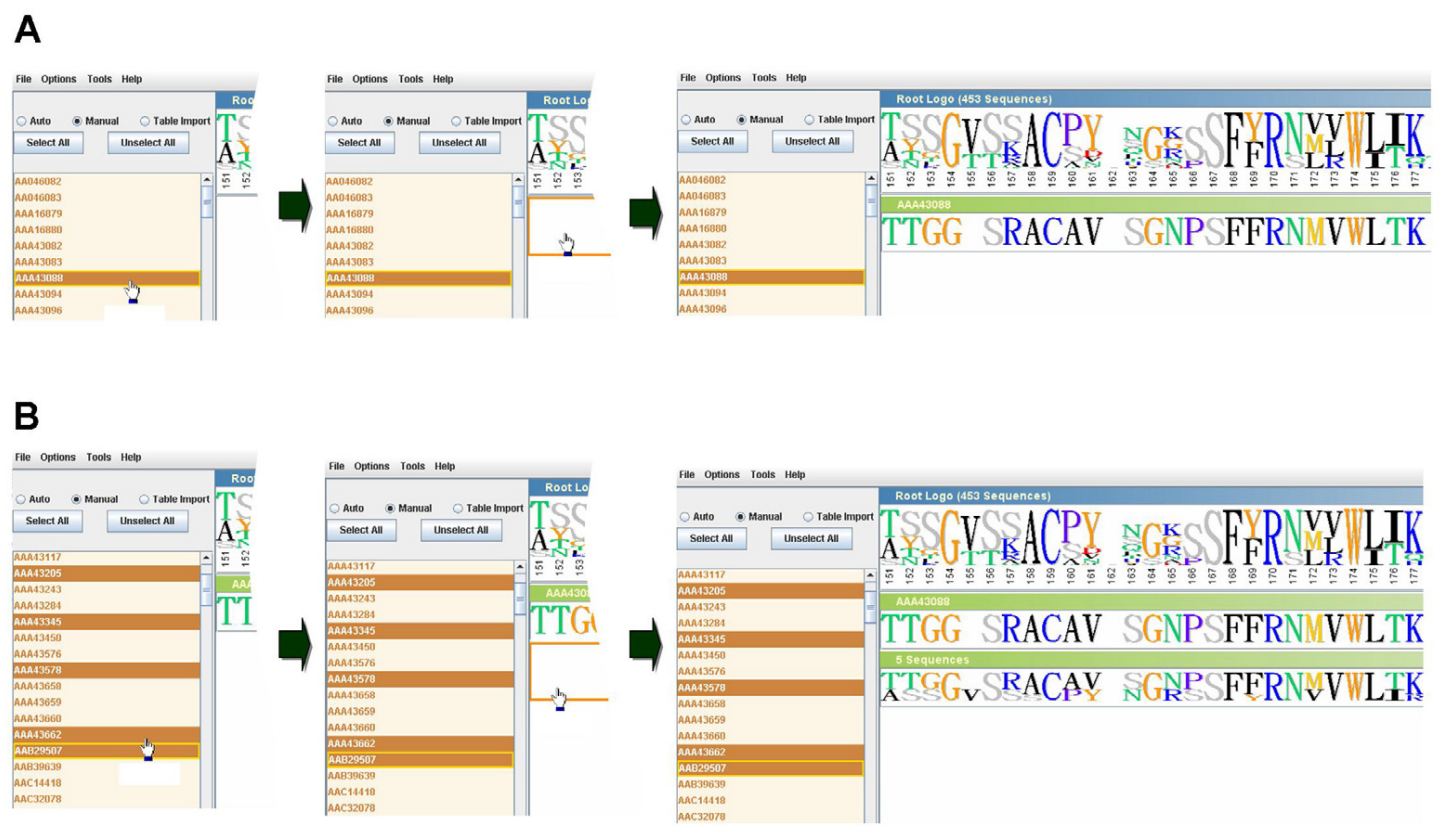
**Generating individual sequence logos of selected sequences**. Individual logos are generated from the selected sequences in the Sequence Pool Panel in Phylogeny/Relationship Viewing Control. A. A single sequence (AAA43088). B. Five selected sequences (AAA43205, AAA43345, AAA43578, AAA43662, and AAB29507).

Moreover, *Phylo-mLogo *also provides a Sequence Pool Panel in Phylogeny/Relationship Viewing Control, which shows the names of all input sequences extracted from the header lines of each sequence in input alignment files. When users want to observe the sequence logo of a single sequence, they can directly click the sequence name and pull it from the Sequence Pool to the expected location in Clade Logos View. The sequence logo will be automatically generated and shown in the location (Fig 2A). If users want to see the sequence logo of a set of designated sequences, they just need to press the *Ctrl *key, simultaneously click these designated sequences, and then pull them to the Clade Logos View. The logos for these selected sequences will be generated immediately (Fig 2B).

In some cases, the user may be interested in only some specific regions. *Phylo-mLogo *provides a function, called Viewing Range Control, to let users select the region of interest and demonstrate only the local sequence logo in the logo viewing panels. Moreover, *Phylo-mLogo *also allows users to select some important, structurally and functionally constrained or informative sites in the aligned sequences and then visualize only the sequence logos of these specific sites. In Site Selection Control, these sites can be selected by the user manually or by built-in automatic calculation which is based on selection of either high frequency bases in low conservation cases or low frequency ones in high conservation cases. Thus, when the length of alignment result is long or the sequences are highly similar or only partially conserved, the user can observe only those informative sites rather than examine the whole alignment.

### Other characteristics of *Phylo-mLogo*

*Phylo-mLogo *is implemented entirely in Java language to ensure portability across major platforms. The execution procedure of the standalone *Phylo-mLogo *is quite straightforward. When launched, the user can load his/her phylogenetic or relationship tree files and the alignment results from the local disk. In Options, there are several options which allow the user to select the display panel, logo type (frequency or entropy), and initial positional index (base index). Furthermore, all graphics of the sequence logos can be exported to portable color image files. For more details of the installation and operations please visit our Website [20].

## Results

In what follows, we will introduce two examples in the study of influenza hemagglutinin (HA) genes and mammalian olfactory receptor (OR) genes to demonstrate how *Phylo-mLogo *can assist users to observe and analyze alignment results of many sequences. The total numbers of alignment sequences in these two examples are 453 and 1425, respectively. The phylogenetic relationships of the aligned sequences are acquired in each example.

### Example 1: 453 avian influenza HA genes

The spread of H5N1 avian influenza from China to Europe has raised a global concern about their potential to infect humans and cause a pandemic. A more comprehensive collection of data and analysis of avian influenza sequences are critically needed for biologists and epidemiologists to find out the virulence and transmissibility of these viruses from avian species to humans. Obenauer *et al*. (2006) established the first large-scale sequencing effort to collect additional genomic data on the avian population of influenza A viruses [3]. They introduced a proteotyping method to identify and number unique amino acid signatures, called proteotypes, for sequences that may or may not be distinguished by branches on a phylogenetic tree. They analyzed eight avian influenza genes and provided the proteograms to demonstrate the amino acid signatures within each clade (Figures S2-S9 in the supplementary material of [3]). Based on the observations, they concluded that the virus families tend to have multiple core conserved genes and that the surface glycoproteins, HA and NA, appear to be more freely exchanged than core proteins because of immune pressure [3].

In this part, we downloaded 437 avian influenza HA genes used for analysis in [3]. Because it was very time consuming to infer the phylogenetic tree of these sequences by MrBayes [3,21], we observed the tree shown in Fig. S6 in [3] directly and constructed their phylogenetic relationship manually. The proteotypes of the analyzed sequences included p1.1, p2.1, p5.1–4, p6.1–6, p8.1, p9.1, p9.2, and p12.1. Based on these proteotypes, we first aligned the sequences of each proteotype and then aligned these proteotypes together, by ClustalW [22]. The total alignment length is 584.

**Figure 3:**
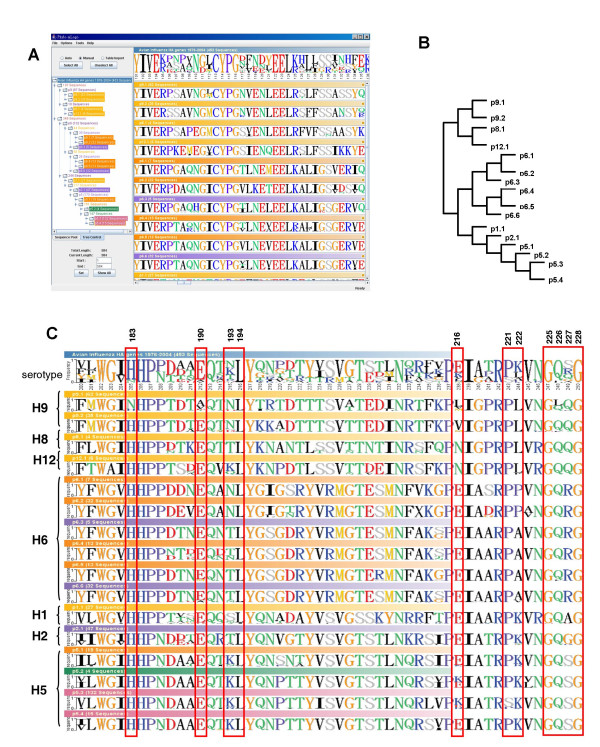
**Multiple-logo visualization of 453 avian HA isolates from 1976 to 2004**. The sequence logos of the root and each clade of 453 avian influenza HA isolates. A. The screenshot of *Phylo-mLogo *result. B. The original phylogenetic tree. C. The important sites located at the receptor binding domain and mapped to the alignment data.

Figure 3A shows the sequence logos and their phylogenetic tree simultaneously. Different from other tools for tree visualization [23], *Plylo-mLogo *displays the phylogenetic tree by using a standard file browser because this representation is more compact than that of the traditional tree visualization of the original phylogenetic tree as shown in Fig. 3B. Thus, the user can click on different clades shown in various background colors, like selecting different folders in a file browser, to visualize the sequence logos of the alignment at different levels.

Stevens *et al*. (2006) listed some conserved residues with the receptor binding domains of H1 and H5 serotypes that are implicated in receptor specificity, amino acid positions 183, 190, 193, 194, 216, 221, 222, and 225–8 [24] to which the corresponding positions in our example are 205, 212, 215, 216, 238, 243, 244, and 247–250, respectively. Then, we compared these sequence logos among different proteotypes. To avoid confusion in this example, we used the original positions shown in Stevens *et al*. (2006). As shown in Fig. 3C, the amino acids at residue sites 194, 225, and 228 are almost conserved across H1, H2, H5, H6, H8, H9, and H12 serotypes. If we only consider H1, H2, and H6, the same clades as H5 [3,25], the amino acids at sites 183, 190, 194, 225, 226, and 228 are almost the same across these serotypes.

Briefly, *Phylo-mLogo *can assist users in comparing and visualizing the changes of polymorphisms and indel events across different clades or subtypes of the alignment of many sequences so that users could speculate possible evolutionary and functional mechanisms and examine their hypotheses further.

### Example 2: 1425 human and mouse functional olfactory receptor genes

Olfactory receptor (OR) genes that encode the proteins used for detecting odor molecules are the largest multigene family in mammals [2,26]. OR genes do not have any intron in the coding regions and the encoded proteins are 300 to 350 amino acids in length. Being a member of the G-protein-coupled receptor (GPCR) superfamily, OR genes include seven transmembrane domains (TM1-TM7, 19–26 amino acid each) interconnected by intracellular and extracellular loops, an extracellular C-terminal chain, and an intracellular N-terminal chain [27,28].

The functional OR genes are separated into class *I *and class *II *[29]. In the human genome, Niimura and Nei (2003) have identified 388 OR genes among which 57 and 331 genes belong to class *I *and class *II*, respectively [1,29]. Moreover, they also identified 19 phylogenetic clades (clades A-S) for human class *II *OR genes and found that many genes belonging to a phylogenetic clade were located in the same genomic cluster [1]. In 2005, they conducted a detailed study of functional OR genes and pseudogenes in mice and identified 1,037 functional genes and 354 pseudogenes. The number of functional genes is 2.7 times greater than that of humans, whereas the number of pseudogenes is slightly smaller in mice than in humans [30].

In this example, we downloaded the whole human and mouse functional OR genes from [1], 1,425 sequences in total, and their detailed annotations. We aligned these sequences by FFT-NS-I [31] and then constructed their neighbor-joining phylogenetic tree by MEGA3 [32]. According to Niimura and Nei's classification [1,30], we also indicated 19 phylogenetic clades (clades A-S) of class *II *OR genes in the final alignment and in the phylogenetic tree.

**Figure 4:**
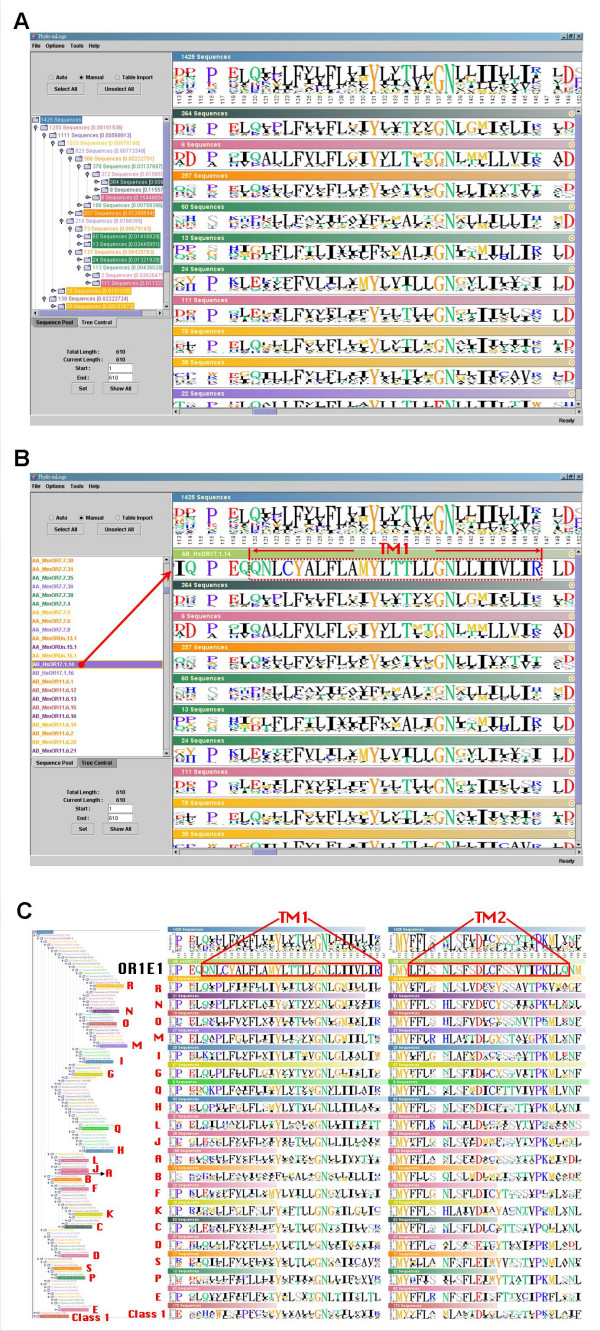
**Multiple-logo visualization of 1425 human and mouse functional OR genes**. The sequence logos of the root and each clade (A-S and class I) of 1425 human and mouse functional OR genes. A. The screenshot of *Phylo-mLogo *result. B. HsOR17.1.14 (as known as OR1E1) was selected as the reference for identifying the TM domains. C. The identified TM1 and TM2 domains and their conservations and variations between different clades.

The phylogenetic relationship of all human and mouse functional OR genes is shown in the left part of Fig. 4A. We selected several major branches which include over ten sequences each, and displayed their corresponding logos shown in the right part of Fig. 4A. We observed that the residues at some sites are highly conserved across different branches but others are not. Some blank regions in the logos show that the columns in these regions across the whole alignment are almost filled with gaps due to some insertions in only a few sequences.

For users to quickly identify and annotate functional regions or specific target sites, we have implemented in *Phylo-mLogo *an operation that allows the user to select some annotated or well-studied sequences in the alignment and to *pull *them from the sequence pool in the Phylogeny/Relationship Viewing Control to Clade Logos View and create new logos for reference. In this example, we selected HsOR17.1.14, also known as OR1E1, as a reference sequence to determine the locations of TM domains in the alignment. We used this sequence because the positions of its TM domains had been identified [33]. Then, we *pulled *the sequence from the sequence pool to the top of Clade Logos View and a new logo of the selected sequence was generated (Fig 4B). Moreover, we also selected 19 phylogenetic clades (clades A-S) of class *II *OR genes according to Niimura and Nei's classification [1,30]. The boundaries of seven TM domains in the alignment can therefore be identified. For example, the start and the end positions of TM1 are 120 and 146 respectively and those of TM2 domains are 164 and 187, respectively (Fig. 4C).

Interestingly, we have found many sites which seem to have undergone positive selection because their amino acids are fixed within some clades but different from those in the other clades. For example, the majority of amino acids at site 120 in TM1 domain in the clades A, D, E, G, H, L, M, N, O, Q, R, and S are Q's (Glutamine, non-charged). But those in clades C,F, and J are E's (Glutamic acid, negatively charged) while those in clade I and in class *I *are both positively charged (K (Lysine) in clade I and H (Histidine) in class *I*). Moreover, the amino acids at site 171 (TM2 domain) in most clades are S's (Serine, polar) but they are A's (Alanine, non-polar) in three clades of class *II*, implying that at these sites in different clades their physico-chemical properties should be different.

To sum up, the residues at a single site which are conserved only within some clades usually could not be identified by a global logo representation. However, these sites are generally considered to have undergone positive selection and possess some kinds of biological meanings. Although the problem of how to identify positively selected sites should be tackled by concrete computational models rather than by visualization, using *Phylo-mLogo*, as the examples show, may allow the user to visually inspect the potential sites in the initial step of sequence analysis as well as to demonstrate computational results in the final stage of representation.

## Discussion

### Different visualization targets for large-scale and many sequence alignment results

To date, deluged by the rate and complexity of completed genomic sequences, the need to align not only longer sequences but also many sequences becomes more urgent. When comparing large-scale sequences, one of the objectives is to identify common conserved regions across species. Usually, the number of sequences is small but the total number of bases could be up to tens of millions [34]. In contrast, when aligning many sequences we focus on finding the heterogeneities of sequences that belong to the same species or population. Thus, the visualization target for large-scale sequence alignments is usually to demonstrate homologous regions and identify their conservations while that for many sequence alignments is to focus on the heterogeneities of the whole alignment and to exhibit common properties of these heterogeneous parts. *Phylo-mLogo *is suitable for users to observe the heterogeneities of an alignment of many sequences as well as to look at the homogeneities of these heterogeneous sites.

### Limitations and further possible enhancements for *Phylo-mLogo*

Generally speaking, *Phylo-mLogo *provides an effective and efficient representation for hierarchical visualization of alignments of many sequences. The proposed representation can help users observe directly the heterogeneities and mutation trends from hundreds or thousands of sequences. However, the sequence-logo-based approach is site independent and does not indicate the relationship between strains. Therefore, combining *Phylo-mLogo *with graphically detailed maps like proteotype [3] and colorful amino acid map in [35] could assist the user to understand the correlative relationship between different strains.

The maximum number of sequences and total length of alignment allowed in *Phylo-mLogo *are dependent on the capacity of internal memory and Java setting of the computer used. In practice, to run *Phylo-mLogo *on a 3GHz Pentium4 PC with 1GB RAM, the largest data set we have tested contains 3,081 H3N2 HA sequences and the length of the alignment is 1,783 bases. In this case, the performance was fine in both loading and manipulation. However, if the user's computer is not as good as what we used, we suggest that the maximum length of alignment had better be less than 1,000 bases in order to obtain a good performance on both sequence logo calculation and displaying speed while the maximum number of sequences could still be in the hundreds.

## Conclusion

Deluged by the increasing number of virus sequences, how to visualize alignment results of many sequences has become a new challenge for computational biologists. In this paper, we have presented a multiple-logo alignment visualization tool. Using *Phylo-mLogo*, the user can visualize the global profile of the whole multiple sequence alignment and to hierarchically examine homologous logos of each clade simultaneously. With *Phylo-mLogo*, the user can symbolically and hierarchically visualize hundreds of aligned sequences simultaneously and easily check the potential sites under different selection pressures, as demonstrated in the analysis of many homologous/orthologous or influenza virus sequences.

## Availability and requirements

Project name: 1. Development of Novel Large-scale Sequence Alignment and Visualization Tools and Their Applications to Bioinformatics

Project home page: 

Operating system(s): Window XP, Sun OS 5.7 Sparc, Mac OS 10.4.2 Tiger, and Linux Fedora Core 3

Programming language: Java

Other requirements: Java 1.4.2 or higher

License: Some restrictions to use by non-academics; free downloads and usage for academics only.

## Abbreviations

**Phylo-mLogo**: Phylogenetic multiple Logos.

**OR**: Olfactory Receptor.

**HA**: Hemagglutinin.

## Authors' contributions

Arthur Chun-Chieh Shih and D.T. Lee contributed the original idea, developed the system organization, and drafted the paper. Chin-Lin Peng took charge of the system development and implementation. Yu-Wei Wu implemented partial code and assisted in some analysis work.
